# Distinct mortality patterns at 0–2 days versus the remaining neonatal period: results from population-based assessment in the Indian state of Bihar

**DOI:** 10.1186/s12916-019-1372-z

**Published:** 2019-07-19

**Authors:** Rakhi Dandona, G. Anil Kumar, Debarshi Bhattacharya, Md. Akbar, Yamini Atmavilas, Priya Nanda, Lalit Dandona

**Affiliations:** 1 Public Health Foundation of India, Sector 44, Institutional Area, Gurugram, National Capital Region India; 2 Institute for Health Metrics and Evaluation, University of Washington, Seattle, USA; 3 Bill & Melinda Gates Foundation, India Country Office, New Delhi, India

**Keywords:** Bihar, Birth asphyxia, Death, India, Preterm, Meningitis, Neonatal mortality, Risk factors, Sepsis, Verbal autopsy

## Abstract

**Background:**

The objectives of this study were to understand the differences in mortality rate, risk factors for mortality, and cause of death distribution in three neonatal age sub-groups (0–2, 3–7, and 8–27 days) and assess the change in mortality rate with previous assessments to inform programmatic decision-making in the Indian state of Bihar, a large state with a high burden of newborn deaths.

**Methods:**

Detailed interviews were conducted in a representative sample of 23,602 live births between January and December 2016 (96.2% participation) in Bihar state. We estimated the neonatal mortality rate (NMR) for the three age sub-groups and explored the association of these deaths with a variety of risk factors using a hierarchical logistic regression model approach. Verbal autopsies were conducted using the PHMRC questionnaire and the cause of death assigned using the SmartVA automated algorithm. Change in NMR from 2011 to 2016 was estimated by comparing it with a previous assessment.

**Results:**

The NMR 0–2-day, 3–7-day, and 8–27-day mortality estimates in 2016 were 24.7 (95% CI 21.8–28.0), 13.2 (11.1 to 15.7), 5.8 (4.4 to 7.5), and 5.8 (4.5 to 7.5) per 1000 live births, respectively. A statistically significant reduction of 23.3% (95% CI 9.2% to 37.3) was seen in NMR from 2011 to 2016, driven by a reduction of 35.3% (95% CI 18.4% to 52.2) in 0–2-day mortality. In the final regression model, the highest odds for mortality in 0–2 days were related to the gestation period of ≤ 8 months (OR 16.5, 95% CI 11.9–22.9) followed by obstetric complications, no antiseptic cord care, and delivery at a private health facility or home. The 3–7- and 8–27-day mortality was driven by illness in the neonatal period (OR 10.33, 95% CI 6.31–16.90, and OR 4.88, 95% CI 3.13–7.61, respectively) and pregnancy with multiple foetuses (OR 5.15, 95% CI 2.39–11.10, and OR 11.77, 95% CI 6.43–21.53, respectively). Birth asphyxia (61.1%) and preterm delivery (22.1%) accounted for most of 0–2-day deaths; pneumonia (34.5%), preterm delivery (33.7%), and meningitis/sepsis (20.1%) accounted for the majority of 3–7-day deaths; meningitis/sepsis (30.6%), pneumonia (29.1%), and preterm delivery (26.2%) were the leading causes of death at 8–27 days.

**Conclusions:**

To our knowledge, this is the first study to report a detailed neonatal epidemiology by age sub-groups for a major Indian state, which has highlighted the distinctly different mortality rate, risk factors, and causes of death at 0–2 days versus the rest of the neonatal period. Monitoring mortality at 0–2 and 3–7 days separately in the traditional early neonatal period of 0–7 days would enable more effective programming to reduce neonatal mortality.

**Electronic supplementary material:**

The online version of this article (10.1186/s12916-019-1372-z) contains supplementary material, which is available to authorized users.

## Background

The slower reduction in neonatal deaths than the reduction in under-5 deaths globally in the past two decades has implications on achieving the Every Newborn target of 10 or fewer neonatal deaths per 1000 live births in every country by 2035 [1, 2]. India accounted for the largest number of under-5 deaths in 2016 at 0·9 million (0·8 to 0·9 million) in 2016, and 54.8% of these were neonatal deaths [2]. As part of the India Newborn Action Plan (INAP), in 2014, the Indian Government adopted a target of < 10 neonatal deaths per 1000 live births by 2030 as a commitment to end preventable newborn deaths [3].

With substantial heterogeneity in the magnitude and pattern of disease burden across the states of India, it is necessary for the cause burden interventions to be tailored at the state level [4, 5]. The Indian state of Bihar is the third most populous Indian state with an estimated population of 110 million in 2016, which contributes substantially to neonatal mortality burden in India [4]. The Bill & Melinda Gates Foundation through the Bihar Technical Support Programme (BTSP) supports the long-term goals of the Government of Bihar to reduce maternal, newborn, and child mortality; improve family planning services; and reduce undernutrition rates in the state [6]. At the start of BTSP in 2011, the neonatal mortality rate (NMR) of 32.2 (95% CI 27.6–36.8) was estimated for Bihar in the baseline survey [7]. In this paper, we report on the change in NMR since 2011, risk factors for neonatal mortality, cause of death distribution, and place of death in 2016 in three neonatal age sub-groups (0–2, 3–7, and 8–27 days) for identifying currently relevant interventions and programme priorities. Importantly, such distinction of the early neonatal mortality (0–7 days) into 0–2- and 3–7-day mortality has not been systematically reported previously from India. By presenting neonatal mortality epidemiology for the age sub-groups, these data are not only programmatically relevant for decision-making in Bihar but also have implications for the INAP in the other Indian states with a high burden of newborn deaths.

## Methods

This study was approved by the Institutional Ethics Committee of the Public Health Foundation of India. All participants provided written informed consent, and for those who could not read or write, the participant information sheet and consent form were explained by the trained interviewer and a thumb impression was obtained.

### Survey design

The methods of this survey are detailed elsewhere, and those relevant to this paper are presented [8]. For this current survey, a sample size of 23,200 live births was estimated to detect a change of 17.5% in neonatal mortality rate in Bihar from 2011 to 2016 with 80% power. The women were selected by a multistage sampling procedure from Bihar state which is divided into 38 districts, each of which is divided into 5–27 blocks, giving a total of 534 blocks in the state. The aim was to obtain a representative sample of women with a birth between January and December 2016 in the state from 50% of the 534 blocks for the study. We first stratified the 534 blocks as with only rural population (70.2%; population range 30,800 to 435,700) and those with both rural and urban population (29.8%; population range 74,380 to 1,771,140). We sampled 267 blocks which included 187 (70%) blocks with only rural population and 80 (30%) blocks with both rural and urban population. Within these 267 blocks, the secondary sampling units (SSUs) were villages in rural areas and urban frame survey blocks in urban areas as defined by the Census 2011 [9]. The SSUs with < 75 households were combined with an adjacent SSU, and the large rural SSUs were split into equal sized segments of 75–100 households using natural boundaries. A total of 1657 SSUs (1475 rural and 182 urban) were sampled in proportion to the number of SSUs in each block, using systematic random sampling without replacement across all the 267 sampled blocks.

### Data collection

Each selected SSU was mapped and all the households (a household was defined as people eating from the same kitchen) enumerated. During the enumeration, trained interviewers documented the birth outcomes among women aged 15–49 years in each household between January and December 2016. Date of birth, sex of the baby born, and whether it was a live birth or stillbirth were documented for each birth. Stillbirths were documented by confirming that the baby did not show any sign of life (did not cry, breathe, and move) in order to differentiate stillbirths and neonatal deaths soon after delivery. We also documented births between January and December 2016 for women who had died during or after giving birth to ensure a robust estimation of total births in this population.

Following enumeration, all women who had reported a birth irrespective of the outcome were eligible for a detailed interview. During the interview, after documenting the background information including socio-demographic characteristics of the participants, questions were asked again to differentiate a stillbirth from a neonatal death that occurred soon after delivery. We documented maternal history during the pregnancy, labour and delivery details, and postnatal care. For babies who had survived for > 2 days after birth, we also documented the history of illness if any and treatment taken for it during the neonatal period. In addition, verbal autopsy (VA) interviews were conducted for all neonatal deaths using the Population Health Metrics Research Consortium shortened VA questionnaire, which includes close-ended questions and an open narrative to ascertain the cause of death [10, 11]. The questionnaires were developed in English and then translated into Hindi (local language), after which these were back-translated into English to ensure the accurate and relevant meaning and intent of the questions. Pilot testing of the questionnaires was carried out and modifications made as necessary. Interviews were captured using the Open Development Kit software in hand-held tablets, and interviews were conducted from March to October 2017. Data entered were scrutinized using the internal consistency checks built in to detect and correct errors using the procedures standardized in the baseline study to meet the data quality. About 30% of the data were collected by the interviewers under direct supervision, and an additional 5% of the interviews were checked by the supervisors by visiting the respondent again. The most frequent discrepancies identified in the initial rounds of data collection were a mismatch of women’s age based on various administrative records, number of antenatal care (ANC) visits, and over-reporting of swelling of the hands and feet under maternal complications. These errors were corrected, and re-training was undertaken for the interviewers.

### Analysis

Before the analysis, we reviewed the narratives of neonates who had died on the day of birth (day 0) to check for possible misreporting between neonatal death and stillbirth. A total of 3 neonatal deaths were reassigned as stillbirth, and no stillbirth was reassigned as a neonatal death. Neonatal death was defined as death within the first 28 days of birth (0–27 days) [12]. We estimated the overall neonatal mortality rate (NMR) per 1000 live births and also in three sub-categories of 0–2 days, 3–7 days, and 8–27 days. NMR was also estimated by sex and place of residence for the state of Bihar in the year 2016. The change in the overall NMR from 2011 to 2016 for Bihar was also estimated [7]. The rates were adjusted for Bihar’s population, and 95% confidence interval (CI) is reported.

#### Risk factors for neonatal mortality

We investigated the association of neonatal deaths for the three age sub-categories with a variety of risk factors, including socio-demographic factors, maternal risk factors, pregnancy, and labour- and delivery-related factors. In addition to these, the risk factors in the postnatal care period were included in models for 3–7-day and 8–27-day deaths. Distribution of and results of unadjusted simple logistic regression are reported for all risk factors that were assessed for all the three models. Furthermore, the associations between the risk factors for neonatal deaths for the three age sub-categories were explored using a hierarchical approach to build the logistic regression model that gave importance to the distal determinants of neonatal mortality [13, 14]. We ran 5 models for 0–2 days and 6 models for 3–7 and 8–27 days deaths with each model adjusted for place of residence and sex of the baby, and each sequential model incorporated variables from the preceding model if *P* was < 0.2 (value for at least one category to be < 0.2 for multiple category variables) [15]. Birthweight was not considered in the adjusted logistic regression as it was not available for 31.4% babies. Wealth index was estimated using the standard questions and methods used in the National Family Health Surveys [16]. For the health facility deliveries, we additionally documented if they had gone to another facility for delivery prior to giving birth in the facility where they gave birth (termed as “referral”). We ran the adjusted models for the three sub-categories for only facility births by including referral delivery as a risk factor. Odds ratio with 95% CI is presented for all models of regression results. All analysis was performed using STATA 13.1 software (Stata Corp., USA).

#### Cause and place of death

As per the Population Health Metrics Research Consortium protocol, the cause of neonatal death was assigned using the validated SmartVA automated algorithm [10, 17, 18]. The cause of neonatal deaths is presented separately for the three sub-categories by the place of delivery. We also report on the place of death for the neonates by place of delivery. For the facility births, we also report the mean days of stay after birth before discharge from the facility.

## Results

A total of 23,602 live births in the year 2016 were identified in 182,486 households (96.2% participation) covering a population of 945,216. A total of 564 neonatal deaths were identified giving an estimated NMR of 24.7 (95% CI 21.8–28.0) per 1000 live births for the state (Table 1). A statistically significant reduction of 23.3% (95% CI − 37.3 to − 9.2) in the overall NMR was documented from 2011 to 2016, an annualized compounded reduction of 5.2% (Table 1). Among the neonatal deaths, 330 (58.5%) were within 0–2 days, 111 (19.7%) in 3–7 days, and 123 (21.8%) in 8–27 days of birth. The NMR by the age sub-categories is shown in Table 1. The largest decline in NMR from 2011 to 2016 was in 0–2-day mortality (− 35.3, 95% CI − 52.2 to − 18.4). A decline of borderline significance at 20.1% was documented for NMR in boys, and the NMR for boys and girls was similar in 2016 (Table 1).

**Table 1:** Neonatal mortality rate (NMR) in 2011 (reference [7]) and 2016 in the Indian state of Bihar by age at death, sex, and place of residence

	Mortality rate per 1000 live births (95% confidence interval)		Percent change in mortality rate from 2011 to 2016 (95% confidence interval)
	2011	2016	
NMR by age at death			
0–2 days	20.4 (16.9 to 23.9)	13.2 (11.1 to 15.7)	− 35.3 (− 52.2 to − 18.4)
3–7 days	6.7 (4.7 to 8.7)	5.8 (4.4 to 7.5)	− 13.4 (− 39.9 to 13.0)
8–27 days	5.0 (3.0 to 6.9)	5.8 (4.5 to 7.5)	16.0 (− 17.0 to 49.0)
0–7 days	27.0 (23.2 to 30.9)	18.9 (16.4 to 21.8)	− 30.0 (− 44.9 to − 15.1)
3–27 days	11.6 (8.4 to 14.9)	11.6 (9.6 to 14.0)	0.0 (− 22.7 to 22.7)
0–27 days	32.2 (27.6 to 36.8)	24.7 (21.8 to 28.0)	− 23.3 (− 37.3 to − 9.2)
NMR by sex			
Boy	32.9 (27.2 to 39.8)	26.3 (22.7 to 30.6)	− 20.1 (− 42.0 to 1.9)
Girl	21.5 (17.4 to 26.7)	23.0 (18.7 to 28.2)	7.0 (− 20.9 to 34.8)
NMR by place of residence			
Rural	32.3 (27.4 to 37.2)	25.3 (22.2 to 28.8)	− 21.7 (− 37.5 to − 5.9)
Urban	25.5 (16.5 to 34.5)	16.0 (9.2 to 27.4)	− 37.3 (− 70.0 to − 4.5)

### Risk factors for neonatal mortality

Of the total live births identified in the enumeration, the detailed interview data were available for 19,877 (84.2% participation) live births including 473 neonatal deaths (83.9% participation). Variations were seen in the distribution and associations of the risk factors using the unadjusted logistic regression for neonatal deaths in the three sub-categories (Table 2). Of all live births, 10,623 (53.5%), 3314 (16.7%), and 5922 (29.8%) were at a public health facility, private health facility, and at home, respectively. Of the 473 neonatal deaths, 216 (45.7%), 121 (25.6%), and 136 (28.7%) were at a public health facility, private health facility, and at home, respectively. The distribution of risk factors by place of delivery for the three sub-categories of neonatal deaths is shown in Additional file 1: Table S1.

**Table 2:** Basic descriptive and results of unadjusted logistic regression with select risk factors for all live births between January and December 2016 and neonatal deaths in 0–2, 3–7, and 8–27 days for those who participated in the detailed interview

Risk factor		All live births, *N* = 19,877 (%)	Neonatal mortality					
			0–2 days, *N* = 280 (%)	Unadjusted OR for 0–2-day death (95% CI)	3–7 days, *N* = 100 (%)	Unadjusted OR for 3–7-day deaths (95% CI)	8–27 days, *N* = 93 (%)	Unadjusted OR for 8–27-day death (95% CI)
Socio-demographic								
Place of residence	Rural	18,126 (91.2%)	265 (94.6%)	1.72 (1.02–2.90)	93 (93.0%)	1.29 (0.60–1.53)	89 (95.7%)	2.17 (0.80–5.92)
	Urban	1751 (8.8%)	15 (5.4%)	1.00	7 (7.0%)	1.00	4 (4.3%)	1.00
Wealth index^a^	Quartile 1	4938 (24.9%)	72 (25.7%)	1.49 (1.03–2.15)	17 (17.2%)	0.86 (0.45–1.65)	31 (33.3%)	2.63 (1.35–5.13)
	Quartile 2	4935 (24.9%)	94 (33.6%)	1.96 (1.38–2.77)	24 (24.2%)	1.22 (0.68–2.22)	25 (26.9%)	2.13 (1.07–4.25)
	Quartile 3	4970 (25.1%)	65 (23.2%)	1.34 (0.92–1.94)	38 (38.4%)	1.92 (1.12–3.30)	25 (26.9%)	2.11 (1.06–4.21)
	Quartile 4	4986 (25.1%)	49 (17.5%)	1.00	20 (20.2%)	1.00	12 (12.9%)	1.00
Sex of baby	Girl	9669 (48.6%)	119 (42.5%)	1.00	48 (48.0%)	1.00	39 (41.9%)	1.00
	Boy	10,208 (51.4%)	161 (57.5%)	1.29 (1.01–1.63)	52 (52.0%)	1.03 (0.69–1.53)	54 (58.1%)	1.32 (0.87–1.99)
Maternal								
Maternal age^b^	15–19 years	924 (4.7%)	20 (7.1%)	1.00	6 (6.0%)	1.00	5 (5.4%)	1.00
	20–24 years	8287 (41.7%)	130 (46.4%)	0.72 (0.45–1.16)	52 (52.0%)	0.96 (0.41–2.24)	39 (41.9%)	0.86 (0.34–2.20)
	25–29 years	7570 (38.1%)	85 (30.4%)	0.51 (0.31–0.84)	28 (28.0%)	0.56 (0.23–1.36)	30 (32.3%)	0.72 (0.28–1.86)
	≥ 30 years	3093 (15.6%)	45 (16.1%)	0.67 (0.39–1.14)	14 (14.0%)	0.69 (0.26–1.80)	19 (20.4%)	1.13 (0.42–3.02)
Any tobacco use ever	Yes	518 (2.6%)	13 (4.6%)	1.84 (1.05–3.23)	5 (5.0%)	2.00 (0.81–4.94)	2 (2.2%)	0.83 (0.20–3.40)
	No	19,359 (97.4%)	267 (95.4%)	1.00	95 (95.0%)	1.00	91 (97.9%)	1.00
Primiparity	Yes	4733 (23.8%)	87 (31.1%)	1.45 (1.12–1.87)	34 (34.0%)	1.66 (1.10–2.52)	22 (23.7%)	1.00 (0.62–1.62)
	No	15,144 (76.2%)	193 (68.9%)	1.00	66 (66.0%)	1.00	71 (76.3%)	1.00
Diabetes mellitus irrespective of pregnancy^c^	Yes	62 (0.3%)	0 (0.0%)	NA	0 (0.0%)	NA	0 (0.0%)	NA
	No	19,452 (99.7%)	275 (100.0%)	NA	99 (100.0%)	NA	91 (100.0%)	NA
Hypertension irrespective of pregnancy^d^	Yes	335 (1.7%)	6 (2.2%)	1.28 (0.57–2.89)	2 (2.0%)	1.19 (0.29–4.84)	1 (1.1%)	0.63 (0.09–4.55)
	No	19,209 (98.3%)	270 (97.8%)	1.00	97 (98.0%)	1.00	91 (98.9%)	1.00
Pregnancy								
At least one antenatal care visit during pregnancy^e^	Yes	16,151 (81.3%)	228 (81.4%)	1.00	74 (74.0%)	1.00	74 (79.6%)	1.00
	No	3725 (18.7%)	52 (18.6%)	0.99 (0.73–1.34)	26 (26.0%)	1.53 (0.98–2.39)	19 (20.4%)	1.12 (0.67–1.85)
Received 2 tetanus toxoid injections during pregnancy^f^	Yes	16,091 (81.2%)	206 (73.6%)	1.00	74 (74.0%)	1.00	70 (75.3%)	1.00
	No	3735 (18.8%)	74 (26.4%)	1.56 (1.19–2.04)	26 (26.0%)	1.53 (0.98–2.39)	23 (24.7%)	1.43 (0.89–2.30)
Consumed iron folic acid tablets during pregnancy^g^	Yes	8007 (40.5%)	105 (37.6%)	1.00	35 (35.0%)	1.00	36 (38.7%)	1.00
	No	11,752 (59.5%)	174 (62.5%)	1.13 (0.89–1.44)	65 (65.0%)	1.27 (0.84–1.92)	57 (61.3%)	1.08 (0.71–1.64)
Pregnancy with multiple foetuses	Yes	336 (1.7%)	21 (7.5%)	4.96 (3.14–7.85)	12 (12.0%)	7.80 (4.13–14.75)	17 (18.3%)	16.04 (9.47–27.19)
	No	19,541 (98.3%)	259 (92.5%)	1.00	88 (88.0%)	1.00	76 (81.7%)	1.00
Hypertension in the last trimester of pregnancy^h^	Yes	504 (2.6%)	14 (5.1%)	2.06 (1.20–3.56)	2 (2.0%)	0.79 (0.19–3.22)	4 (4.6%)	1.84 (0.67–5.02)
	No	19,908 (97.4%)	261 (94.9%)	1.00	97 (98.0%)	1.00	84 (95.5%)	1.00
Malaria in last 3 months of pregnancy^i^	Yes	449 (2.3%)	4 (1.5%)	0.62 (0.23–1.67)	3 (3.0%)	1.32 (0.42–4.19)	1 (1.1%)	0.47 (0.07–3.37)
	No	19,064 (97.7%)	272 (98.6%)	1.00	96 (97.0%)	1.00	90 (98.9%)	1.00
Syphilis during pregnancy^j^	Yes	83 (0.4%)	1 (0.4%)	0.86 (0.12–6.17)	0 (0.0%)	NA	0 (0.0%)	NA
	Do not know	1151 (5.8%)	17 (6.1%)	1.05 (0.64–1.72)	8 (8.0%)	1.41 (0.68–2.92)	6 (6.5%)	1.12 (0.49–2.57)
	No	18,642 (93.8%)	262 (93.6%)	1.00	92 (92.0%)	1.00	87 (93.6%)	1.00
Fever in the last 3 months of pregnancy^k^	Yes	3547 (18.0%)	33 (11.9%)	0.61 (0.42–0.88)	18 (18.0%)	0.99 (0.60–1.65)	17 (18.3%)	1.01 (0.60–1.71)
	No	16,130 (82.0%)	244 (88.1%)	1.00	82 (82.0%)	1.00	76 (81.7%)	1.00
Convulsion in last 3 months of pregnancy^l^	Yes	2215 (11.3%)	36 (13.0%)	1.18 (0.83–1.68)	10 (10.2%)	0.89 (0.46–1.72)	7 (7.5%)	0.64 (0.29–1.38)
	No	17,333 (88.7%)	240 (87.0%)	1.00	88 (89.8%)	1.00	86 (92.5%)	1.00
Mother is informed the baby was not growing adequately inside the womb^m^	Yes	679 (3.5%)	18 (6.5%)	1.33 (0.42–4.18)	3 (3.1%)	0.91 (0.29–2.89)	8 (8.7%)	2.71 (1.31–5.63)
	No	18,959 (96.5%)	261 (93.6%)	1.00	93 (96.9%)	1.00	84 (91.3%)	1.00
Gestation period	≤ 8 months	477 (2.4%)	73 (26.1%)	16.75 (12.61–22.27)	14 (14.0%)	7.98 (4.49–14.15)	16 (17.2%)	10.57 (6.11–18.29)
	> 8 months	19,400 (97.6%)	207 (73.9%)	1.00	86 (86.0%)	1.00	77 (82.8%)	1.00
Labour								
Mother had come for delivery earlier but was asked to come later for delivery (deferred delivery^n^	Yes	160 (0.8%)	3 (1.1%)	1.33 (0.42–4.18)	3 (3.1%)	3.97 (1.24–12.66)	4 (4.5%)	5.98 (2.17–16.51)
	No	19,515 (99.2%)	277 (98.9%)	1.00	94 (96.9%)	1.00	85 (95.5%)	1.00
Spontaneous labour^o^	Yes	14,637 (74.1%)	189 (68.2%)	1.00	65 (67.0%)	1.00	62 (69.7%)	1.00
	No	5120 (25.9%)	88 (31.8%)	1.34 (1.04–1.73)	32 (33.0%)	1.42 (0.93–2.17)	27 (30.3%)	1.25 (0.80–1.97)
Foul-smelling liquor^p^	Yes	976 (4.9%)	20 (7.2%)	1.50 (0.95–2.38)	8 (8.1%)	1.71 (0.83–3.53)	7 (7.7%)	1.62 (0.75–3.52)
	No	18,768 (95.1%)	258 (92.8%)	1.00	91 (91.9%)	1.00	84 (92.3%)	1.00
Labour for more than 12 h^q^	Yes	3040 (15.4%)	47 (17.0%)	1.13 (0.82–1.54)	17 (17.2%)	1.14 (0.68–1.93)	16 (17.6%)	1.18 (0.69–2.02)
	No	16,720 (84.6%)	230 (83.0%)	1.00	82 (82.8%)	1.00	75 (82.4%)	1.00
Delivery								
Place of delivery^r^	Public	10,623 (53.5%)	120 (42.9%)	1.00	54 (54.0%)		42 (45.2%)	1.00
	Private	3314 (16.7%)	76 (27.1%)	2.05 (1.54–2.75)	22 (22.0%)	1.32 (0.81–2.18)	23 (24.7%)	1.78 (1.07–2.97)
	Home	5922 (29.8%)	84 (30.0%)	1.26 (0.95–1.67)	24 (24.0%)	0.80 (0.49–1.29)	28 (30.1%)	1.20 (0.74–1.94)
Mother had gone to a health facility for delivery but delivered in another facility (referred delivery)^s^	Yes	781 (5.6%)	29 (14.9%)	3.02 (2.02–4.51)	6 (7.9%)	1.48 (0.64–3.43)	8 (12.5%)	2.49 (1.18–5.24)
	No	13,154 (94.4%)	166 (85.1%)	1.00	70 (92.1%)	1.00	56 (87.5%)	1.00
Vaginal delivery^t^	Yes	17,867 (90.0%)	243 (87.1%)	0.75 (0.53–1.07)	82 (82.0%)	0.50 (0.30–0.84)	81 (89.0%)	0.89 (0.46–1.73)
	No	1991 (10.0%)	36 (12.9%)	1.00	18 (18.0%)	1.00	10 (11.0%)	1.00
Push/pull done during delivery by the health provider^u^	Yes	965 (5.0%)	40 (14.6%)	3.38 (2.40–4.76)	15 (15.2%)	3.57 (2.06–6.22)	4 (4.4%)	0.92 (0.34–2.51)
	No	18,533 (95.1%)	234 (85.4%)	1.00	84 (84.9%)	1.00	87 (95.6%)	1.00
Entangled cord around the baby’s neck^v^	Yes	761 (3.8%)	21 (7.5%)	2.04 (1.30–3.21)	7 (7.0%)	1.89 (0.87–4.10)	4 (4.3%)	1.15 (0.42–3.14)
	Do not know	1748 (8.8%)	20 (7.2%)	0.83 (0.53–1.32)	7 (7.0%)	0.81 (0.37–1.74)	8 (8.6%)	0.98 (0.47–2.02)
	No	17,351 (87.4%)	238 (85.3%)	1.00	86 (86.0%)	1.00	81 (87.1%)	1.00
Breech position of the baby^w^	Yes	633 (3.2%)	30 (11.0%)	3.82 (2.59–5.63)	6 (6.1%)	2.00 (0.87–4.60)	7 (7.5%)	2.55 (1.17–5.53)
	No	18,888 (96.8%)	243 (89.0%)	1.00	93 (93.9%)	1.00	86 (92.5%)	1.00
Antiseptic cord care	Yes	4966 (25.0%)	31 (11.1%)	1.00	23 (23.0%)	1.00	20 (21.5%)	1.00
	No	11,901 (59.9%)	183 (65.4%)	2.49 (1.70–3.64)	61 (61.0%)	1.12 (0.69–1.81)	62 (66.7%)	1.31 (0.79–2.17)
	Do not know	3010 (15.1%)	66 (23.6%)	3.57 (2.32–5.48)	16 (16.0%)	1.17 (0.62–2.21)	11 (11.8%)	0.92 (0.44–1.93)
Birthweight of the baby (kg)^x^	≥ 2.5	11,401 (57.9%)	57 (37.8%)	1.00	38 (43.2%)	1	37 (41.6%)	1
	< 2.5	2037 (10.4%)	27 (17.9%)	2.67 (1.69–4.24)	19 (21.6%)	2.84 (1.63–4.93)	30 (33.7%)	4.66 (2.87–7.56)
	Never weighted	4704 (23.9%)	53 (35.1%)	2.18 (1.52–3.12)	23 (26.1%)	1.32 (0.80–2.17)	18 (20.2%)	1.10 (0.64–1.91)
	Do not know if weighted	1547 (7.9%)	14 (9.3%)	1.74 (0.98–3.11)	8 (9.1%)	1.39 (0.66–2.95)	4 (4.5%)	0.74 (0.27–2.07)
Postnatal care								
Baby put in an incubator after birth^y^	Yes	984 (5.1%)			31 (31.0%)	8.68 (5.65–13.33)	25 (26.9%)	7.27 (4.57–11.55)
	No	18,482 (94.9%)			69 (69.0%)	1.00	68 (73.1%)	1.00
Received delayed bathing, skin-to-skin care, and immediate breastfeeding^z^	Yes	2124 (10.9%)			6 (6.3%)	1.00	7 (7.8%)	1.00
	No	17,435 (89.1%)			89 (93.7%)	1.81 (0.79–4.14)	83 (92.2%)	1.45 (0.67–3.14)
Received postnatal care within 1 week of birth	Yes	7009 (35.8%)			41 (41.0%)	1.00	35 (37.6%)	1.00
	No	12,588 (64.2%)			59 (59.0%)	0.80 (0.54–1.19)	58 (62.4%)	0.92 (0.60–1.40)
Illness and treatment during neonatal period^aa^	No illness	15,862 (90.0%)			25 (25.5%)	1.00	39 (41.9%)	1.00
	Untreated illness	1381 (7.0%)			38 (38.8%)	17.92 (10.79–29.78)	22 (23.7%)	6.75 (3.99–11.41)
	Ill and out-patient treatment sought	1936 (9.9%)			12 (12.2%)	3.95 (1.98–7.88)	14 (15.1%)	2.97 (1.61–5.48)
	Illness that required hospitalization	409 (2.1%)			23 (23.5%)	37.75 (21.24–67.09)	18 (19.4%)	19.81 (11.23–34.96)

Data not available for ^a^48 (0.24%) in 0–2 and 3–7 days and 47 (0.24%) in 8–27 days; ^b^3 (0.02%) in all; ^c^363 (1.83%), 358 (1.83%), and 357 (1.83%) in 0–2, 3–7, and 8–27 days, respectively; ^d^333 (1.68%), 329 (1.68%), and 328 (1.68%) in 0–2, 3–7, and 8–27 days, respectively; ^e^1 (0.01%) in all; ^f^51 (0.26%) in all; ^g^118 (0.59%) in 0–2 and 117 (0.60%) in each 3–7 and 8–27 days; ^h^275 (1.38%), 270 (1.38%), and 269 (1.38%) in 0–2, 3–7, and 8–27 days, respectively; ^i^364 (1.83%), 360 (1.84%), and 359 (1.84%) in 0–2, 3–7, and 8–27 days, respectively; ^j^1 (0.01%) in all; ^k^200 (1.01%) in 0–2 and 197 (1.01%) in 3–7 and 8–27 days; ^l^329 (1.66%), 325 (1.66%), and 323 (1.66%) in 0–2, 3–7, and 8–27 days, respectively; ^m^239 (1.20%), 238 (1.22%), and 234 (1.20%) in 0–2, 3–7, and 8–27 days, respectively; ^n^202 (1.02%) in 0–2 and 3–7 days and 199 (1.02%) in 8–27 days; ^o^120 (0.60%), 117 (0.60%), and 114 (0.59%) in 0–2, 3–7, and 8–27 days, respectively; ^p^133 (0.67%), 131 (0.67%), and 130 (0.67%) in 0–2, 3–7, and 8–27 days, respectively; ^q^117 (0.59%), 114 (0.59%), and 113 (0.59%) in 0–2, 3–7, and 8–27 days, respectively; ^r^18 (0.19%) in all; ^s^data shown only for women who delivered in a health facility; data not available for 2 (0.01%) in 0–2 and 1 (0.01%) in 3–7 and 8–27 days; ^t^19 (0.10%) in 0–2 and 18 (0.10%) in 3–7 and 8–27 days; ^u^379 (1.91%), 373 (1.90%), and 372 (1.91%) in 0–2, 3–7, and 8–27 days, respectively; ^v^17 (0.09%) in 0–2 and 16 (0.08%) in 3–7 and 8–27 days; ^w^356 (1.79%), 349 (1.79%), and 348 (1.79%) in 0–2, 3–7, and 8–27 days, respectively; ^×^188 (0.95%), 59 (0.30%), and 47 (0.24%) in 0–2, 3–7, and 8–27 days, respectively; ^y^131 (0.67%) in both; ^z^38 (0.19%) and 33 (0.17%) in 3–7 and 8–27 days, respectively; and ^aa^9 (0.05%) and 7 (0.04%) in 3–7 and 8–27 days, respectively

*OR* odds ratio, *CI* confidence interval

After adjusting for the place of residence and sex of the neonate in the final sequential logistic regression model for mortality in 0–2 days (Table 3), gestation period of ≤ 8 months had the highest odds for mortality in this period (OR 16.52, 95% CI 11.93–22.88) followed by obstetric complications including push/pull during the delivery by health provider (OR 2.88, 95% CI 1.93–4.32), breech position of the baby (OR 2.70, 95% CI 1.72–4.25), entangled cord around the baby’s neck (OR 2.02, 95% CI 1.23–3.34), and pregnancy with multiple foetuses (OR 2.31, 95% CI 1.30–4.11). Not receiving antiseptic cord care at birth (OR 2.40, 95% CI 1.60–3.59) and delivery either at private health facility or at home (OR 1.58, 95% CI 1.21–2.06) also had significantly higher odds of death in 0–2 days. The neonates belonging to any wealth index quartile other than the highest quartile 4 (OR 1.83, 95% CI 1.28–2.59), primi babies (OR 1.44, 95% CI 1.07–1.93), babies of women who did not receive tetanus toxoid (TT) immunization during pregnancy (OR 1.54, 95% CI 1.14–2.09), and boy babies (OR 1.32, 95% CI 1.02–1.71) also had significantly higher odds of death in 0–2 days.

**Table 3:** Results of sequential multiple logistic regression models for the association of neonatal death within 0–2 days of birth with select risk factors in the Indian state of Bihar

Risk factors	Adjusted odds ratio for neonatal death in 0–2 days (95% confidence interval)				
	Model 1*	Model 2*	Model 3*	Model 4*	Model 5*
Rural place of residence	1.45 (0.85–2.47)	1.40 (0.82–2.39)	1.50 (0.85–2.65)	1.56 (0.89–2.74)	1.43 (0.79–2.56)
Boy baby	1.29 (1.01–1.63)	1.27 (1.00–1.62)	1.37 (1.07–1.77)	1.35 (1.05–1.73)	*1.32 (1.02–1.71)*
Wealth index quartile					
I	1.41 (0.98–2.05)	1.50 (1.02–2.18)	2.02 (1.35–3.02)	1.86 (1.25–2.77)	*2.01 (1.31–3.07)*
II	1.85 (1.30–2.64)	1.89 (1.32–2.71)	2.30 (1.56–3.37)	2.23 (1.53–3.25)	*2.41 (1.61–3.60)*
III	1.28 (0.88–1.87)	1.31 (0.90–1.93)	1.45 (0.97–2.16)	1.33 (0.89–1.99)	1.46 (0.96–2.22)
IV	1.00	1.00	1.00	1.00	1.00
Maternal age					
15–19 years		1.00	1.00	1.00	
20–24 years		0.81 (0.50–1.33)	0.81 (0.49–1.35)	0.94 (0.56–1.60)^‡^	
25–29 years		0.64 (0.37–1.11)	0.69 (0.39–1.21)	0.79 (0.45–1.41)^‡^	
≥ 30 years		0.81 (0.45–1.46)	0.76 (0.41–1.40)	0.81 (0.43–1.53)^‡^	
Any tobacco use ever		1.75 (0.99–3.10)	1.73 (0.95–3.14)	1.79 (0.99–3.26)	1.64 (0.90–2.99)
Primiparity		1.37 (1.01–1.86)	1.41 (1.02–1.96)	1.43 (1.03–1.98)	*1.44 (1.07–1.93)*
Maternal history of high blood pressure irrespective of pregnancy		1.28 (0.57–2.90)^‡^			
No maternal antenatal care visit during pregnancy			1.00 (0.71–1.39)^‡^		
Mother did not receive 2 tetanus toxoid injections during pregnancy			1.52 (1.13–2.05)	1.49 (1.11–2.00)	*1.54 (1.14–2.09)*
Mother did not consume iron folic acid tablets during pregnancy			0.99 (0.76–1.28)^‡^		
Pregnancy with multiple foetuses			2.92 (1.72–4.95)	3.33 (1.96–5.64)	*2.31 (1.30–4.11)*
Maternal hypertension in the last trimester of pregnancy			2.00 (1.06–3.77)	1.82 (0.99–3.36)	1.34 (0.68–2.66)
Mother had malaria in the last trimester of pregnancy			0.66 (0.23–1.90)^‡^		
Diagnosed with syphilis during pregnancy					
No			1.00		
Yes			1.43 (0.19–10.66)^‡^		
Do not know			1.06 (0.63–1.80)^‡^		
Mother had a fever in the last 3 months of pregnancy			0.57 (0.39–0.85)	0.56 (0.38–0.83)	*0.49 (0.32–0.74)*
Mother had convulsions in the last 3 months of pregnancy			0.94 (0.64–1.38)^‡^		
Mother is informed the baby was not growing adequately inside the womb			1.44 (0.81–2.54^‡^)		
Gestation period					
≤ 8 months			16.74 (12.26–22.86)	17.03 (12.50–23.20)	*16.52 (11.93–22.88)*
> 8 months			1.00	1.00	1.00
Deferred delivery				0.84 (0.25–2.83)^‡^	
Spontaneous labour				1.30 (0.99–1.71)	*1.38 (1.03–1.84)*
Foul-smelling liquor				1.46 (0.88–2.42)	1.12 (0.64–1.96)
Labour for more than 12 h				1.07 (0.76–1.51)^‡^	
Place of delivery					
Public facility					1.00
Private facility					*1.88 (1.31–2.70)*
Home					*1.39 (1.02–1.90)*
Vaginal delivery					1.26 (0.79–2.00)
Push/pull done during delivery by the health provider					*2.88 (1.93–4.32)*
Entangled cord around the baby’s neck					
No					1.00
Yes					*2.02 (1.23–3.34)*
Do not know					0.60 (0.34–1.07)
Breech presentation of the baby					*2.70 (1.72–4.25)*
Antiseptic cord care					
Yes					1.00
No					*2.40 (1.60–3.59)*
Do not know					*3.85 (2.44–6.10)*

*Model adjusted for sex of the baby and place of residence

^‡^*P ≥* 0.2 and hence excluded from the sequential model

For mortality at 3–7 days (Table 4), illness in the neonatal period (OR 10.33, 95% CI 6.31–16.90) had the highest odds of death followed by pregnancy with multiple foetuses (OR 5.15, 95% CI 2.39–11.10), gestation period of ≤ 8 months (OR 3.76, 95% CI 1.87–7.58), and babies who were put in an incubator after birth (OR 3.28, 95% CI 1.88–5.75). Pregnancies with no antenatal care (OR 1.98, 95% CI 1.18–3.34), without TT immunization during pregnancy (OR 1.94, 95% CI 1.19–3.16), and those belonging to wealth index quartile 3 (OR 2.71, 95% CI 1.42–5.16) also had significantly higher odds of death in 3–7 days. For deaths at 8–27 days (Table 5), pregnancy with multiple foetuses (OR 11.77, 95% CI 6.43–21.53), illness in the neonatal period (OR 4.88, 95% CI 3.13–7.61), and gestation period of ≤ 8 months (OR 6.15, 95% CI 3.18–11.87) had significantly higher odds of death. The babies who were put in an incubator after birth (OR 2.72, 95% CI 1.49–4.96) and those belonging to any wealth index quartile other than quartile 4 (OR 3.04, 95% CI 1.53–6.04) also had significantly higher odds of death at 8–27 days.

**Table 4:** Results of sequential multiple logistic regression models for the association of neonatal deaths in 3–7 days after birth with select risk factors in the Indian state of Bihar

Risk factors	Adjusted odds ratio for neonatal death in 3–7 days (95% confidence interval)					
	Model 1*	Model 2*	Model 3*	Model 4*	Model 5*	Model 6*
Rural place of residence	1.21 (0.55–2.67)	1.15 (0.52–2.54)	1.10 (0.49–2.47)	1.30 (0.55–3.10)	1.56 (0.61–4.03)	0.79 (0.34–1.82)
Boy baby	1.06 (0.71–1.57)	1.09 (0.73–1.62)	1.13 (0.75–1.70)	1.19 (0.78–1.80)	1.20 (0.79–1.82)	1.04 (0.67–1.62)
Wealth index quartile						
I	0.84 (0.43–1.62)	0.92 (0.47–1.80)	0.89 (0.44–1.79)	0.73 (0.34–1.57)	0.91 (0.42–1.96)	1.18 (0.54–2.55)
II	1.19 (0.65–2.18)	1.29 (0.69–2.39)	1.20 (0.63–2.28)	1.41 (0.74–2.71)	1.55 (0.79–3.05)	1.57 (0.77–3.21)
III	1.87 (1.08–3.25)	2.03 (1.16–3.57)	1.94 (1.09–3.47)	2.23 (1.22–4.04)	2.43 (1.31–4.52)	*2.71 (1.42–5.16)*
IV	1.00	1.00	1.00	1.00	1.00	1.00
Maternal age						
15–19 years		1.00				
20–24 years		1.07 (0.45–2.55)^‡^				
25–29 years		0.74 (0.28–1.92)^‡^				
≥ 30 years		0.89 (0.32–2.53)^‡^				
Any tobacco use ever		2.23 (0.89–5.59)	1.72 (0.62–4.79)^‡^			
Null parity		1.38 (0.84–2.26)^‡^				
Maternal history of high blood pressure irrespective of pregnancy		1.20 (0.29–4.90)^‡^				
No maternal antenatal care visit during pregnancy			1.47 (0.89–2.44)	1.87 (1.15–3.03)	1.88 (1.14–3.10)	*1.98 (1.18–3.34)*
Mother did not receive 2 tetanus toxoid injections during pregnancy			1.58 (0.99–2.52)	1.45 (0.90–2.33)	1.61 (1.00–2.59)	*1.94 (1.19–3.16)*
Mother did not consume iron folic acid tablets during pregnancy			1.11 (0.72–1.71)^‡^			
Pregnancy with multiple foetuses			6.13 (3.11–12.07)	6.35 (3.16–12.75)	5.28 (2.50–11.15)	*5.15 (2.39–11.10)*
Maternal hypertension in the last trimester of pregnancy			0.75 (0.17–3.19)^‡^			
Mother had malaria in the last trimester of pregnancy			1.54 (0.46–5.14)^‡^			
Diagnosed with syphilis during pregnancy						
No			1.00			
Yes			NA			
Do not know			1.60 (0.76–3.36)^‡^			
Mother had a fever in the last 3 months of pregnancy			1.07 (0.63–1.82)^‡^			
Mother had convulsions in the last 3 months of pregnancy			0.95 (0.49–1.87)^‡^			
Mother is informed the baby was not growing adequately inside the womb			0.75 (0.23–2.48)^‡^			
Gestation period						
8 months or less			6.99 (3.76–13.00)	6.13 (3.20–11.76)	6.40 (3.31–12.38)	*3.76 (1.87–7.58)*
> 8 months			1.00	1.00	1.00	1.00
Deferred delivery				2.68 (0.77–9.29)	3.04 (0.90–10.31)	2.00 (0.53–7.53)
Spontaneous labour				1.45 (0.93–2.28)	1.31 (0.83–2.08)^‡^	
Foul-smelling liquor				1.58 (0.74–3.39)^‡^		
Labour for more than 12 h				1.05 (0.60–1.83)^‡^		
Place of delivery						
Public facility					1.00	
Private facility					0.90 (0.47–1.70)^‡^	
Home					0.88 (0.52–1.49)^‡^	
Vaginal delivery					0.54 (0.27–1.09)	0.58 (0.31–1.07)
Push/pull done during delivery by the health provider					2.55 (1.33–4.86)	1.81 (0.93–3.51)
Entangled cord around the baby’s neck						
No					1.00	
Yes					1.47 (0.63–3.47)^‡^	
Do not know					0.95 (0.43–2.09)^‡^	
Breech presentation of the baby					0.95 (0.36–2.46)^‡^	
Antiseptic cord care						
Yes					1.00	
No					0.99 (0.60–1.64)^‡^	
Do not know					1.20 (0.62–2.31)^‡^	
Baby put in the incubator						
No						1.00
Yes						*3.28 (1.88–5.75)*
Baby received delayed bathing, skin-to-skin care, and immediate breastfeeding						1.25 (0.54–2.93)
Received postnatal care within 1 week of birth						1.48 (0.91–2.39)
Illness and treatment in the neonatal period						
No illness						1.00
Untreated illness						*15.71 (9.10–27.13)*
Ill and out-patient treatment sought						*3.82 (1.84–7.92)*
Illness that required hospitalization						*22.34 (11.21–44.52)*

*Model adjusted for sex of the baby and place of residence

^‡^*P ≥* 0.2 and hence excluded from the sequential model

**Table 5:** Results of sequential multiple logistic regression models for the association of neonatal deaths in 8–27 days after birth with select risk factors in the Indian state of Bihar

Risk factors	Adjusted odds ratio for neonatal death in 8–27 days (95% confidence interval)					
	Model 1*	Model 2*	Model 3*	Model 4*	Model 5*	Model 6*
Rural place of residence	1.64 (0.59–4.56)	1.64 (0.59–4.56)	1.64 (0.57–4.71)	1.74 (0.59–5.17)	1.62 (0.57–4.62)	1.33 (0.47–3.81)
Boy baby	1.33 (0.88–2.00)	1.30 (0.86–1.96)	1.47 (0.95–2.27)	1.44 (0.93–2.24)	1.53 (0.99–2.37)	1.31 (0.84–2.05)
Wealth index quartile						
I		2.49 (1.26–4.92)	2.89 (1.38–6.05)	3.47 (1.63–7.36)	4.08 (1.88–8.87)	*3.77 (1.73–8.23)*
II		1.92 (0.94–3.88)	2.47 (1.17–5.22)	2.89 (1.34–6.20)	3.31 (1.51–7.23)	*2.88 (1.32–6.29)*
III		2.00 (1.00–4.03)	2.48 (1.20–5.13)	2.47 (1.15–5.31)	2.63 (1.22–5.67)	*2.93 (1.37–6.26)*
IV		1.00	1.00	1.00	1.00	1.00
Maternal age						
15–19 years		1.00				
20–24 years		1.13 (0.39–3.24)^‡^				
25–29 years		0.92 (0.30–2.82)^‡^				
≥ 30 years		1.39 (0.43–4.47)^‡^				
Any tobacco use ever		0.69 (0.17–2.81)^‡^				
Null parity		1.02 (0.58–1.79)^‡^				
Maternal history of high blood pressure irrespective of pregnancy		0.64 (0.09–4.62)^‡^				
No maternal antenatal care visit during pregnancy			1.22 (0.71–2.09)^‡^			
Mother did not receive 2 tetanus toxoid injections during pregnancy			1.29 (0.78–2.15)^‡^			
Mother did not consume iron folic acid tablets during pregnancy			0.96 (0.61–1.49)^‡^			
Pregnancy with multiple foetuses			9.88 (5.41–18.03)	11.89 (6.63–21.34)	10.57 (5.86–19.07)	*11.77 (6.43–21.53)*
Maternal hypertension in the last trimester of pregnancy			1.43 (0.42–4.93)^‡^			
Mother had malaria in the last trimester of pregnancy			0.35 (0.05–2.70)^‡^			
Diagnosed with syphilis during pregnancy						
No			1.00			
Yes			NA			
Do not know			1.07 (0.45–2.52)^‡^			
Mother had a fever in the last 3 months of pregnancy			0.88 (0.50–1.57)^‡^			
Mother had convulsions in the last 3 months of pregnancy			0.61 (0.27–1.37)^‡^			
Mother is informed the baby was not growing adequately inside the womb			2.68 (1.22–5.89)	2.00 (0.89–4.48)	2.06 (0.92–4.58)	1.52 (0.68–3.39)
Gestation period						
8 months or less			10.76 (5.86–19.78)	11.80 (6.45–21.60)	10.68 (5.81–19.63)	*6.15 (3.18–11.87)*
> 8 months			1.00	1.00	1.00	1.00
Deferred delivery				2.20 (0.58–8.31)^‡^		
Spontaneous labour				1.24 (0.77–2.01)^‡^		
Foul-smelling liquor				1.50 (0.66–3.39)^‡^		
Labour for more than 12 hours				0.97 (0.53–1.77)^‡^		
Place of delivery						
Public facility					1.00	1.00
Private facility					1.86 (1.01–3.44)	1.38 (0.76–2.52)
Home					1.14 (0.69–1.87)	1.35 (0.79–2.33)
Vaginal delivery					1.08 (0.48–2.42)^‡^	
Push/pull done during delivery by the health provider					0.55 (0.17–1.81)^‡^	
Entangled cord around the baby’s neck						
No					1.00	
Yes					1.06 (0.38–2.98)^‡^	
Do not know					1.23 (0.58–2.61)^‡^	
Breech presentation of the baby					1.58 (0.68–3.69)^‡^	
Antiseptic cord care						
Yes					1.00	
No					1.31 (0.78–2.21)^‡^	
Do not know					1.06 (0.50–2.26)^‡^	
Baby put in the incubator						
No						1.00
Yes						*2.72 (1.49–4.96)*
Baby received delayed bathing, skin-to-skin care, and immediate breastfeeding						1.02 (0.46–2.27)
Received postnatal care within 1 week of birth						1.43 (0.85–2.42)
Illness and treatment in the neonatal period						
No illness						1.00
Untreated illness						*5.50 (3.11–9.74)*
Ill and out-patient treatment sought						*2.83 (1.49–5.37)*
Illness that required hospitalization						*12.16 (6.21–23.79)*

*Model adjusted for sex of the baby and place of residence

^‡^*P≥*0.2 and hence excluded from the sequential model

As illness during the neonatal period had the highest odds of death at 3–27 days, illness symptoms and treatment sought were explored among the neonates who had survived for 3 days or more (Additional file 3: Figure S1). High fever (10.1%) was the most commonly reported symptom followed by loss of interest in breastfeeding (4.4%), difficult/rapid breathing (4.1%), and baby is cold to touch (4.1%). Among those with reported illness, most treatments were sought for high fever (73%) and yellowing of the skin (69.3%) and the least for baby drowsy/difficult to awaken (24.8%) and discoloration of the skin around the cord (23.9%).

On considering referred deliveries as a risk factor for the facility births (data not shown), referral delivery was significantly associated with mortality at 0–2 days (OR 2.35, 95% CI 1.44–3.83) with no significant change in the results of the previous model. No significant association of referred deliveries was found with mortality at 3–7 and 8–27 days.

### Place of death

**Fig. 1:**
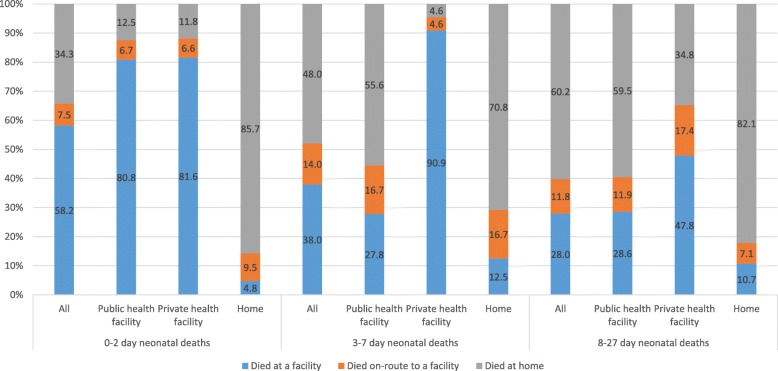
Distribution of place of death by place of delivery for neonatal deaths in the Indian state of Bihar

Overall, 227 (48%), 46 (9.7%), and 200 (42.3%) of the neonates had died at a facility, en route to a facility, and at home, respectively. By place of delivery, 57.4%, 76.9%, and 7.4% of the births at a public facility, private facility, and at home had died at a facility, respectively. Distribution of place of death varied by age at death and place of delivery (Fig. 1). In 0–2-day deaths, majority of the facility deaths were also facility births (81.1%) and the majority of home deaths were home births (85.7%). In 3–7- and 8–27-day deaths, the proportion of facility deaths was higher for the private facility births as compared with the public facility and home births.

Among the 13,961 (70.2%) health facility births, the mean days of stay at the facility for neonates before discharge were significantly lower at the public as compared with the private facilities both for those who survived and those who died during the neonatal period (Additional file 2: Table S2). Importantly, 62.5% and 48.7% of the neonates who had died at 0–2 days were discharged alive from the public and private facility after birth, respectively. The mean stay at the facility for these neonates was 4.2 times shorter in the public as compared with the private facility after birth. A significantly higher proportion of neonates who died at 3–7 days were discharged alive from the public (94.4%) as compared with private facilities (63.6%), with the mean days of stay at the facility before discharge lower in the former. A similar proportion of neonates who died at 8–27 days were discharged alive from the public (90.5%) as compared with the private facility (95.7%).

### Causes of death for neonatal mortality

**Fig. 2:**
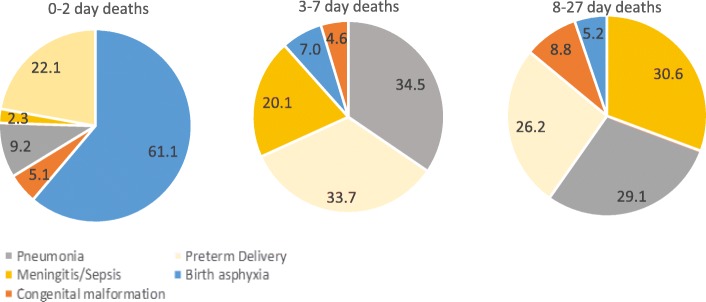
Distribution of causes of death using verbal autopsy interviews by the three age sub-groups in the Indian state of Bihar

The distribution of causes of death varied for the three sub-categories of neonatal deaths (Fig. 2). Birth asphyxia (61.1%) was the leading cause of death in 0–2 days followed by preterm delivery (22.1%); pneumonia (34.5%), preterm delivery (33.7%), and meningitis/sepsis (20.1%) in 3–7 days; and meningitis/sepsis (30.6%) and pneumonia (29.1%) followed by preterm delivery (26.2%) in 8–27 days.

Varying patterns for the cause of death were seen based on place of delivery in the age sub-groups (Additional file 4: Figure S2). The proportion of preterm delivery deaths was the least (16.6%) and birth asphyxia was the most (67.5%) for home deliveries as compared with the facility deliveries in 0–2-day deaths (24.5% and 58.4%, respectively). In 3–7-day deaths, the proportion of meningitis/sepsis was higher in public sector (24%) and home deliveries (22.6%) as compared to the private sector deliveries (7.9%) whereas the proportion of deaths due to congenital malformation (11.7%) and birth asphyxia (11.1%) was the highest in private sector deliveries. Preterm delivery accounted for 52.2% of the causes of death at home deliveries in 3–7-day deaths. Similar varying patterns for the causes of death were seen based on the place of delivery, particularly for preterm delivery and meningitis/sepsis, in 8–27-day deaths.

## Discussion

The estimated NMR in Bihar was 24.7 deaths per 1000 live births in 2016, a significant reduction of 23% from 2011, which was driven by the reduction in the 0–2-day mortality with no significant change in 3–27 days mortality. The presentation of the risk factors and cause of death based on the age of neonate at the death and place of delivery and the place of death yield useful insights for programming to reduce neonatal mortality further in this population. Importantly, the splitting of the early neonatal age group of 0–7 days into 0–2 and 3–7 days highlights substantial differences not only in the mortality rate but also in the risk factors and causes of death between these sub-groups. These findings offer critical insights for better targeting of interventions in the early neonatal age groups and for monitoring and planning reduction in NMR. From the programme perspective, the fact that 0–2-day mortality is reducing and is still more than half of NMR is an opportunity to enhance this reduction further. In addition, more effective focus on reducing 3–7- and 8–27-day mortality is also needed based on its major risk factors and causes.

The 0–2-day NMR was 2.3 times higher than the 3–7-day NMR in 2016. Even with a decline of 35% between 2011 and 2016, 0–2 days mortality accounted for 53.4% of the total neonatal mortality in 2016. Though this study was not designed to capture the possible reasons for 0–2-day NMR decline, the decrease corroborates with the increased coverage of facility deliveries, public sector in particular, in this population between 2011 and 2016 [7], and improvements in infection control and intrapartum practices in high delivery volume public sector facilities undertaken through the nurse mentoring in the BTSP in Bihar [19]. With regard to the risk factors, a gestation period of ≤ 8 months was associated with neonatal deaths across the three age sub-groups; however, the highest risk was documented for 0–2-day deaths. It is estimated that 60% of all preterm births are in South Asia and sub-Saharan Africa, and these births account for 80% of neonatal deaths in these regions [20–22]. A massive 82.8% of the neonatal deaths in India in 2017 could be attributed to low birth weight and short gestation [23], and its contribution to DALYs is higher in Bihar than the national average [4]. As the pregnancy length was captured in months in our study, it is difficult to comment on whether the premature babies were very or moderately preterm [24]. Preterm labour is considered to be a syndrome initiated by multiple mechanisms, and more understanding in the classification of preterm and of its risk factors is needed in this population, both for spontaneous and provider-initiated preterm births [25–27]. The obstetric complications including breech position presentation, pregnancy with multiple foetuses, cord around the baby’s neck, and deliveries with “push and forceful pull” were associated with 0–2 days mortality, but only pregnancy with multiple foetuses was associated with 3–27 days mortality in this study. In addition to poor skills of staff providing emergency obstetric care in India [28–32], several other related health system barriers have been identified for emergency obstetric care, including the inadequacy of staff, equipment, and accountability [33]. The need for improving the emergency neonatal care is further highlighted by the causes of death in 0–2 days with birth asphyxia accounting for 61% and preterm births for 22% of these deaths. The proportion of deaths attributed to birth asphyxia decreased thereafter, and home births had a slightly higher proportion of these deaths in 0–2 days. Birth asphyxia and preterm births are known to account for the majority of deaths in the early neonatal period [22, 34]. Emergency neonatal care that includes management of asphyxia and extra care for low birth weight and preterm babies is one of the most cost-effective intervention packages to save newborns [35]. The shortage of appropriately trained human resources is recognized as a major bottleneck by INAP to improve quality of care for complicated deliveries [3, 36]. In addition, these data also point to equip human resources with the ability to manage birth asphyxia and preterm through prompt identification, stabilization, and appropriate referral [22], and to improve the understanding of neonatal mortality by gestation months to develop facility- and community-based interventions to better manage the preterm newborns.

The absence of antiseptic cord care, delivering in the private sector and at home, being a male, and being the firstborn were associated with a higher risk of death in 0–2 days period but not in the 3–27 days period. Despite the dry cord care recommendation for all births by the Ministry of Health in India [37], antiseptic for cord care was reported in one quarter of the deliveries in this study which was associated with a lower 0–2-day mortality. We have previously reported similar results from this population highlighting that the application of readily available gentian violet on the cord after birth in less developed settings should be assessed further for its potential beneficial influence on neonatal mortality [38]. The 0–2-day mortality was higher both in the private facility and home births as compared with the public facility births. In the last 5 years, the proportion of home births has declined significantly by 20.5% but that of private facility births has remained unchanged in this population [7]. The home deliveries were predominately conducted by untrained birth attendants such as untrained Dai, family member, and friends rather than a skilled birth attendant in this population [8]. Ways to promote safe facility deliveries in women preferring home delivery by addressing relevant barriers for facility delivery and improving birth preparedness for those preferring home delivery are needed to address 0–2-day neonatal mortality [39–41]. Interpretation of a higher 0–2-day mortality in the private facilities should take into account the context of referred deliveries in this population and the finding of higher mortality in deliveries with obstetric complications and among the referred deliveries [8]. It is well known that the private sector provides most of the emergency obstetric care in India and also serves as a referral facility for the public sector for complicated deliveries [42, 43]. To address the neonatal mortality in such deliveries, BTSP is exploring a partnership with the private sector for improved quality of care, which can be considered under the INAP as well [3, 44]. Ensuring quality of care in a private sector is important with the increasing involvement of the private sector in the universal health coverage agenda of the Government of India [45].

A 20.1% decline of borderline statistical significance in overall NMR was seen in boys between 2011 and 2016. Boys accounted for 55.6% of all neonatal deaths in 2016, and the NMR was similar for both sexes in 2016. A higher risk of neonatal mortality in boys accounted for by biological differences between boys and girls that favour girl survival during the neonatal period is known [46] and is seen in 0–2-day mortality in this population. As this study was not designed to capture the reasons for NMR decline by sex, this finding needs to be looked into further given India’s obsession with boy child to explore if access to better services or treatment factors accounts for this differential decline by sex in this population [47].

The 3–27 days mortality accounted for 47% of all neonatal mortality in 2016 in this population with illness during the neonatal period being its most significant predictor. This is also reflected in the cause of death distribution with pneumonia and meningitis/sepsis accounting for a major proportion of these deaths in addition to preterm births. Though home visitation during the neonatal period by a health worker to check on the baby for early identification of danger signs and prompt treatment and referral is meant to be part of the routine health system [48], its coverage is quite poor [49, 50]. In this population, only one third of the neonates who survived > 2 days received a postnatal care visit in the first week of birth. The latest DHS survey 2015–2016 reported coverage of postnatal care visit in 24 h after birth at 11.2% for Bihar [16]. Babies who were sick and not treated and those who needed hospitalization suggesting increased severity of illness were significantly more likely to die during this period. The preterm deaths continued to account for 26–33% of deaths in 3–27 days, thereby highlighting the need for effective postnatal care in these babies. In addition, the illness- and treatment-seeking patterns presented for various illness symptoms in the community offer insight into the challenges at the community level for early identification and treatment of sick babies. In addition to targeting the families of low birth weight and premature babies for extra care, the INAP could also include babies who are put in the incubator post-delivery for extra postnatal care attention and consider intervening at the community level to improve early identification of danger signs and prompt treatment and referral [3, 49].

Two doses of TT immunization are among the established and cost-effective interventions to save newborn babies [35]. Pregnancies without the TT doses were significantly more likely to result in 0–2- and 3–7-day mortality in this study. Over the last 5 years, a significant decline of 12.1% in TT immunization coverage during pregnancy was seen in this population [7]. This drop in coverage reflects poor quality of ANC services as TT doses are administered under the ANC interventions. Furthermore, pregnancies with no ANC were associated with a higher risk of 3–7-day mortality in this study. Improving ANC services is imperative to reduce adverse pregnancy outcomes as the specific interventions delivered in this period are meant to prevent or identify and treat infections, pregnancy-induced conditions, and undernutrition [36, 51, 52]. The possible reasons for the inadequacy of assessment or knowledge of the risk factors such as syphilis, diabetes, and hypertension in pregnant women have been previously reported by us from this population [8], highlighting that neonatal mortality cannot be addressed fully unless the quality of ANC services improves to deliver maternal interventions. In this direction, the Government of India has recently taken steps to address the coverage and quality of ANC services to pregnant women through a specific programme [53].

In order to address neonatal mortality comprehensively, it is important to understand where the babies die in addition to the cause of death and risk factors for neonatal mortality. Overall, 42% of the neonates died at home, and babies who were born at home were also most likely to die at home irrespective of the age at death. This finding is of concern as it highlights not only the limited access to emergency obstetric care but also the limited access to illness treatment during the neonatal period for this sub-group of babies. Targeting the barriers to uptake of institutional delivery and understanding health-seeking behaviour practices in this group would be important to reduce neonatal mortality in home deliveries [54–58]. A significantly different pattern was seen for facility births by age sub-groups. Though the majority of 0–2-day deaths occurred at the facility irrespective of the type of health facility, the proportion of public sector births dying at the facility was significantly lower than the private sector births for 3–27-day deaths. Also, as the mean days of stay were significantly lower in the former than the latter, further in-depth exploration would be useful to understand the context of duration of facility stay in relation to the delivery complications or other barriers such as costs and quality of care in addition to the treatment-seeking behaviour to develop strategies to reduce neonatal mortality [59].

There are some limitations to the study findings. As is the case with surveys, the findings should be interpreted within the context of recall bias of the respondent. The gestational age was captured in months instead of weeks as the pregnancy length in India is reported in months. The last menstrual period forms the basis for most and is considered a reliable estimate for measuring gestational age in both developing and developed country settings [60, 61]. Strengthening of the numerator and denominator for neonatal mortality estimation by documenting all in/out-migration among the reproductive age women with pregnancy outcome in the period of interest and differentiation of stillbirth from immediate neonatal death from enumeration through the analysis is a major strength. Presenting the findings by the three age sub-groups supports the need for a continuum of care as the core principle to address the further decline in neonatal mortality [62]. We used a hierarchical approach to the risk factor analysis that gave importance to distal determinants of neonatal mortality and have presented results in a manner that allows for better understanding of the association of various risk factors of interest [13].

With the variations highlighted in the decline in NMR, and in drivers and causes of death of neonatal mortality by the age sub-groups, we strongly recommend that the INAP monitor neonatal mortality in these age sub-groups by splitting the early neonatal period into 0–2 and 3–7 days. Such monitoring will also ensure that every newborn is taken into account through a framework of integrated packages of service delivery for the health of mothers and newborn babies along the continuum of care [1, 62]. Our data on neonatal mortality epidemiology by the age sub-groups can be used to specifically adapt the evidence-based intervention framework to deliver across the continuum of care from pregnancy to the neonatal period [62]. Furthermore, as the neonates belonging to wealth index strata other than the highest were more likely to die in this population, it is imperative to address inequity across the continuum of care by reaching every newborn to further reduce neonatal mortality [63].

## Conclusions

Given the variations in the risk factors and causes of death in the three age sub-groups as shown in this study, it would be useful to rethink the current categorization of neonatal age groups and split the early neonatal group of 0–7 days into 0–2 days and 3–7 days groups to plan interventions and monitor mortality changes more specifically in these two periods.

## Additional files

Additional file 1: **Table S1.** Basic descriptive during labour and delivery for all live births between January and December 2016 and for neonatal deaths in 0–2, 3–7, and 8–27 days by the place of delivery for those who participated in the detailed interview. (DOCX 37 kb)

Additional file 2: **Table S2.** Survival status at discharge post-birth and mean days of facility stay for neonates born at a facility in the Indian state of Bihar. (DOCX 22 kb)

Additional file 3: **Figure S1.** Distribution of illness symptoms (not mutually exclusive) and treatment sought for that symptom among newborns who survived 3 days or more in the Indian state of Bihar. (DOCX 23 kb)

Additional file 4: **Figure S2.** Distribution of causes of death using verbal autopsy interviews by place of delivery for the three age sub-groups in the Indian state of Bihar. (DOCX 66 kb)

## Data Availability

All data generated or analysed during this study are included in this published article and its additional information files.

## Abbreviations

ANC: Antenatal care
BTSP: Bihar Technical Support Programme
CI: Confidence interval
INAP: Indian Newborn Action Plan
NMR: Neonatal mortality rate
TT: Tetanus toxoid
