# Supplementary material for: Distinct mortality patterns at 0–2 days versus the remaining neonatal period: results from population-based assessment in the Indian state of Bihar
Source: BMC Med. 2019 Jul 19;17:140. doi: 10.1186/s12916-019-1372-z (PMC6639919; doi:10.1186/s12916-019-1372-z)
Supplement: Supplementary file 1 — Table S1. Basic descriptive during labour and delivery for all live births between January and December 2016 and for neonatal deaths in 0–2, 3–7, and 8–27 days by the place of delivery for those who participated in the detailed interview. (DOCX 37 kb) [file 12916_2019_1372_MOESM1_ESM.docx]

**Additional Table 1**. Basic descriptive during labour and delivery for all live births between January and December 2016 and for neonatal deaths in 0-2, 3-7 and 8-27 days by the place of delivery for those who participated in the detailed interview.

| **Risk factors** | | **Public facility** | | | | **Private facility** | | | | **Home** | | | |
| --- | --- | --- | --- | --- | --- | --- | --- | --- | --- | --- | --- | --- | --- |
|  |  | **All live births N=10,623 (%)** | **0-2 day neonatal death**  **N=120 (%)** | **3-7 days neonatal death**  **N=54 (%)** | **8-27 days neonatal death**  **N=42 (%)** | **All live births N=3,314 (%)** | **0-2 day neonatal death**  **N=76 (%)** | **3-7 days neonatal death**  **N=22 (%)** | **8-27 days neonatal death**  **N=23 (%)** | **All live births N=5,922 (%)** | **0-2 day neonatal death**  **N=84 (%)** | **3-7 days neonatal death**  **N=24 (%)** | **8-27 days neonatal death**  **N=28 (%)** |
| Gestation period (months)^*^ | <8 | 184  (1.7%) | 21 (17.5%) | 5  (9.3%) | 5  (11.9%) | 180 (5.4%) | 21 (27.6%) | 4  (18.2%) | 5  (21.7%) | 113  (1.9%) | 31 (36.9%) | 5  (20.8%) | 6  (21.4%) |
|  | >8 | 10,439 (98.3%) | 99 (82.5%) | 49 (90.7%) | 37 (88.1%) | 3,134 (94.6%) | 55 (72.4%) | 18 (81.8%) | 18 (78.3%) | 5,809 (98.1%) | 53 (63.1%) | 19 (79.2%) | 22 (78.6%) |
| Mother had come for delivery earlier but was asked to come later for delivery (deferred delivery) ^†^ | Yes | 68  (0.7%) | 1  (0.8%) | 1  (1.9%) | 0 | 68  (2.1%) | 2  (2.6%) | 1  (4.8%) | 2  (8.7%) | 23  (0.4%) | 0 | 1  (4.2%) | 2  (7.7%) |
|  | No | 10,454 (99.4%) | 119 (99.2%) | 51 (98.1%) | 40  (100%) | 3,205 (97.9%) | 74 (97.4%) | 20 (95.2%) | 21 (91.3%) | 5,839 (99.6%) | 84  (1.4%) | 23 (95.8%) | 24 (92.3%) |
| Spontaneous labour^‡^ | Yes | 7,596 (71.7%) | 76 (65.0%) | 35 (64.8%) | 29 (70.7%) | 1,971 (60.7%) | 43 (56.6%) | 9  (45.0%) | 11 (52.4%) | 5,058 (85.8%) | 70 (83.3%) | 21 (91.3%) | 22 (81.5%) |
|  | No | 2,998 (28.3%) | 41 (35.0%) | 19 (35.2%) | 12 (29.3%) | 1,277 (39.3%) | 33 (43.4%) | 11 (55.0%) | 10 (47.6%) | 839 (14.2%) | 14 (16.7%) | 2  (8.7%) | 5  (18.5%) |
| Foul smelling liquor^§^ | Yes | 526  (5.0%) | 8  (6.7%) | 5  (9.3%) | 4  (9.8%) | 178 (5.4%) | 8  (10.7%) | 3  (13.6%) | 1  (4.6%) | 272  (4.6%) | 4  (4.8%) | 0 | 2  (7.1%) |
|  | No | 10,028 (95.0%) | 111 (93.3%) | 49 (90.7%) | 37 (90.2%) | 3,108 (94.6%) | 67 (89.3%) | 19 (86.4%) | 21 (95.5%) | 5,614 (95.4%) | 80 (95.2%) | 23  (100%) | 26 (92.9%) |
| Labour for more than 12 hours^#^ | Yes | 1,652 (15.7%) | 20 (17.0%) | 11 (20.8%) | 6  (15.0%) | 696 (21.1%) | 19 (25.0%) | 4  (18.2%) | 6  (26.1%) | 689 (11.7%) | 8  (1.2%) | 2  (8.3%) | 4  (14.3%) |
|  | No | 8,906 (84.4%) | 98 (83.1%) | 42 (79.3%) | 34 (85.0%) | 2,601 (78.9%) | 57 (75.0%) | 18 (81.8%) | 17 (73.9%) | 5,198 (88.3%) | 75 (90.4%) | 22 (91.7%) | 24 (85.7%) |
| Vaginal delivery^**^ | Yes | 10,040 (94.6%) | 108 (90.8%) | 50 (92.6%) | 38 (92.7%) | 1,897 (57.3%) | 51 (67.1%) | 8  (36.4%) | 15 (68.2%) | 5,913 (100%) | 84  (100%) | 24  (100%) | 28  (100%) |
|  | No | 577  (5.4%) | 11  (9.2%) | 4  (7.4%) | 3  (7.3%) | 1,413 (42.7%) | 25 (32.9%) | 14 (63.6%) | 7  (31.8%) | 0 | 0 | 0 | 0 |
| Push/pull done during delivery by the health provider ^††^ | Yes | 544  (5.2%) | 24 (20.2%) | 10 (18.5%) | 1  (2.4%) | 291 (9.3%) | 11 (15.3%) | 3  (14.3%) | 2  (9.1%) | 129  (2.2%) | 5  (6.0%) | 2  (8.3%) | 1  (3.6%) |
|  | No | 9,917 (94.8%) | 95 (79.8%) | 44 (81.5%) | 40 (97.6%) | 2,843 (90.7%) | 61 (84.7%) | 18 (85.7%) | 20 (90.9%) | 5,757 (97.8%) | 78 (94.0%) | 22 (91.7%) | 27 (96.4%) |
| Entangled cord around baby’s neck^‡‡^ | Yes | 367  (3.5%) | 8  (6.7%) | 5  (9.3%) | 2  (4.8%) | 134 (4.1%) | 7  (9.2%) | 0 | 0 | 260  (4.4%) | 6  (7.1%) | 2  (8.3%) | 2  (7.1%) |
|  | Don’t know | 973  (9.2%) | 11  (9.2%) | 2  (3.7%) | 3  (7.1%) | 390 (11.8%) | 7  (9.2%) | 3  (13.6%) | 3  (13.0%) | 385  (6.5%) | 2  (2.4%) | 2  (8.3%) | 2  (7.1%) |
|  | No | 9,273 (87.4%) | 100 (84.0%) | 47 (87.0%) | 37 (88.1%) | 2,786 (84.2%) | 62 (81.6%) | 19 (86.4%) | 20 (87.0%) | 5,274 (89.1%) | 76 (90.5%) | 20 (83.3%) | 24 (85.7%) |
| Breech position of the baby^§§^ | Yes | 324  (3.1%) | 14 (11.8%) | 5  (9.4%) | 3  (7.1%) | 148 (4.7%) | 8  (11.1%) | 1  (4.6%) | 1  (4.4%) | 160  (2.7%) | 8  (9.8%) | 0 | 3  (10.7%) |
|  | No | 10,168 (96.9%) | 105 (88.2%) | 48 (90.6%) | 39 (92.9%) | 2,986 (95.3%) | 64 (88.9%) | 21 (95.5%) | 22 (95.7%) | 5,717 (97.3%) | 74 (90.2%) | 24  (100%) | 25 (89.3%) |
| Antiseptic cord care^##^ | Yes | 2,672 (25.2%) | 12 (10.0%) | 14 (25.9%) | 10 (23.8%) | 926 (27.9%) | 7  (9.2%) | 3  (13.6%) | 3  (13.0%) | 1,326 (23.0%) | 12 (14.3%) | 6  (25.0%) | 7  (25.0%) |
|  | No | 6,158 (58.0%) | 76 (63.3%) | 31 (57.4%) | 29 (69.1%) | 1,644 (49.6%) | 45 (59.2%) | 12 (54.6%) | 14 (60.9%) | 4,089 (69.1%) | 62 (73.8%) | 18 (75.0%) | 19 (67.9%) |
|  | Don’t know | 1,793 (16.9%) | 32 (26.7%) | 9  (16.7%) | 3  (7.1%) | 744 (22.5%) | 24 (31.6%) | 7  (31.8%) | 6  (26.1%) | 471  (8.0%) | 10 (11.9%) | 0 | 2  (7.1%) |
| Birthweight of the baby (kilograms)^***^ | ≥2.5 | 7,989 (75.8%) | 36 (53.7%) | 27 (56.3%) | 25 (65.8%) | 2,316 (70.9%) | 20 (48.8%) | 10 (58.8%) | 8  (34.8%) | 1,088 (18.5%) | 1  (2.3%) | 1  (4.4%) | 4  (14.3%) |
|  | <2.5 | 1,429  (13.6%) | 16  (23.9%) | 12  (25.0%) | 11  (30.0%) | 442 (13.5%) | 8  (19.5%) | 4  (23.5%) | 14 (60.9%) | 164  (2.8%) | 3  (7.0%) | 3  (13.0%) | 5  (17.9%) |
|  | Never weighted | 322  (3.1%) | 8  (11.9%) | 5  (10.4%) | 0 | 279 (8.5%) | 8  (19.5%) | 0 | 0 | 4,100 (69.9%) | 37 (86.1%) | 18 (78.3%) | 18 (64.3%) |
|  | Don’t know if weighted | 797  (7.6%) | 7  (10.5%) | 4  (8.3%) | 2  (5.3%) | 231 (7.1%) | 5  (12.2%) | 3  (17.7%) | 1  (4.4%) | 515  (8.8%) | 2  (4.7%) | 1  (4.4%) | 1  (3.6%) |

*Chi-square test for significance: p<0.001 for all in 0-2 day and 8-27 days; p<0.001 for public facility and home delivery and p=0.004 for private facility in 3-7 days

^†^Data not available for 101, 41 and 60 births for 0-2 day in public, private and home delivery, respectively, Chi-square test for significance: p=0.797 for public facility, 0.732 for private facility and 0.562 for home delivery; data not available for 101, 41 and 60 births for 3-7 days in public facility, private facility and home delivery, respectively, Chi-square test for significance: p= 0.248 for public facility, 0.383 for private facility and 0.003 for home delivery; data not available for 99, 40 and 60 births for 8-28 days in public facility, private facility and home delivery, respectively, Chi-square test for significance: p=0.612 for public facility, 0.024 for private facility and <0.001 for home delivery

^‡^Data not available for 29, 66 and 25 births for 0-2 day in public facility, private facility and home delivery, respectively, Chi-square test for significance: p=0.103 for public facility, 0.459 for private facility and 0.519 for home delivery; data not available for 26, 63 and 25 births for 3-7 days in public facility, private facility and home delivery, respectively, Chi-square test for significance: p=0.254 for public facility, 0.147 for private facility and 0.449 for home delivery; data not available for 26, 64 and 24 births for 8-28 days in public facility, private facility and home delivery, respectively, Chi-square test for significance: p=0.878 for public facility, 0.423 for private facility and 0.521 for home delivery

^§^Data not available for 69, 28 and 36 births for 0-2 day in public facility, private facility and home delivery, respectively, Chi-square test for significance: p=0.381 for public facility, 0.042 for private facility and 0.951 for home delivery; data not available for 68, 27 and 36 births for 3-7 days in public facility, private facility and home delivery, respectively, Chi-square test for significance: p= 0.303 for public facility, 0.743 for private facility and 0.604 for home delivery; data not available for 68, 27 and 35 births for 8-27 days in public facility, private facility and home delivery, respectively, Chi-square test for significance: p= 0.154 for public facility, 0.884 for private facility and 0.527 for home delivery

^#^Data not available for 65, 17 and 35 births for 0-2 day in public facility, private facility and home delivery, respectively, Chi-square test for significance: p=0.695 for public facility, 0.401 for private facility and 0.556 for home delivery; data not available for 63, 17 and 34 births for 3-7 days in public facility, private facility and home delivery, respectively, Chi-square test for significance: p=0.048 for public facility, 0.842 for private facility and 0.965 for home delivery; data not available for 62, 17 and 34 births for 8-27 days in public facility, private facility and home delivery, respectively, Chi-square test for significance: p=0.916 for public facility, 0.551 for private facility and 0.676 for home delivery

^**^Data not available for 6, 4 and 9 births for 0-2 day in public facility, private facility and home delivery, respectively, Chi-square test for significance: p=0.065 for public facility and 0.081 for private facility; data not available for 5, 4 and 9 births for 3-7 days in public facility, private facility and home delivery, respectively, Chi-square test for significance: p=0.511 for public facility and 0.049 for private facility; data not available for 5, 4 and 9 births for 8-27 days in public facility, private facility and home delivery, respectively, Chi-square test for significance: p= 0.582 for public facility and 0.297 for private facility

^††^Data not available for 162, 180 and 36 births for 0-2 day in public facility, private facility and home delivery, respectively, Chi-square test for significance: p<0.001 for public facility, 0.076 for private facility and 0.016 for home delivery; data not available for 161, 176 and 35 births for 3-7 days in public facility, private facility and home delivery, respectively, Chi-square test for significance: p<0.001 for public facility, 0.412 for private facility and 0.035 for home delivery; data not available for 161, 175 and 35 births for 8-27 days in public facility, private facility and home delivery, respectively, Chi-square test for significance: p=0.457 for public facility, 0.998 for private facility and 0.590 for home delivery

^‡‡^Data not available for 10, 4 and 3 births for 0-2 day in public facility, private facility and home delivery, respectively, Chi-square test for significance: p=0.145 for public facility, 0.060 for private facility and 0.156 for home delivery; data not available for 9, 4 and 3 births for 3-7 days in public facility, private facility and home delivery, respectively, Chi-square test for significance: p=0.026 for public facility, 0.624 for private facility and 0.582 for home delivery; data not available for 9, 4 and 3 births for 8-27 days in public facility, private facility and home delivery, respectively, Chi-square test for significance: p=0.807 for public facility, 0.617 for private facility and 0.755 for home delivery

^§§^Data not available for 131, 180 and 45 births for 0-2 day in public facility, private facility and home delivery, respectively, Chi-square test for significance: p<0.001 for public facility and home delivery, 0.01 for private facility; data not available for 130, 176 and 43 births for 3-7 days in public facility, private facility and home delivery, respectively, Chi-square test for significance: p=0.006 for public facility, 0.995 for private facility and 0.420 for home delivery; data not available for 129, 176 and 43 births for 8-27 days in public facility, private facility and home delivery, respectively, Chi-square test for significance: p=0.108 for public facility, 0.959 for private facility and 0.007 for home delivery

^##^ Chi-square test for significance: p<0.001 for public facility, 0.001 for private facility and 0.092 for home delivery in 0-2 day; Chi-square test for significance: p=0.995 for public facility, 0.255 for private facility and 0.356 for home delivery in 3-7 day; Chi-square test for significance: p=0.195 for public facility, 0.256 for private facility and 0.966 for home delivery in 8-27 day

^***^  Data not available for 86, 46 and 55 births for 0-2 day in public facility, private facility and home delivery, respectively, Chi-square test for significance: p<0.001 for public facility and home delivery, 0.002 for private facility; data not available for 33, 11 and 14 births for 3-7 days in public facility, private facility and home delivery, respectively, Chi-square test for significance: p=0.001 for public facility, 0.013 for private facility and 0.231 for home delivery; data not available for 27, 6 and 13 births for 8-27 days in public facility, private facility and home delivery, respectively, Chi-square test for significance: p<0.001 for all
