# Supplementary material for: Distinct mortality patterns at 0–2 days versus the remaining neonatal period: results from population-based assessment in the Indian state of Bihar
Source: BMC Med. 2019 Jul 19;17:140. doi: 10.1186/s12916-019-1372-z (PMC6639919; doi:10.1186/s12916-019-1372-z)
Supplement: Supplementary file 2 — Table S2. Survival status at discharge post-birth and mean days of facility stay for neonates born at a facility in the Indian state of Bihar. (DOCX 22 kb) [file 12916_2019_1372_MOESM2_ESM.docx]

**Additional Table 2**. Survival status at discharge post birth and mean days of facility stay for neonates born at a facility in the Indian state of Bihar.

|  | **Health facility birth** | **Public health facility birth** | **Private health facility birth** |
| --- | --- | --- | --- |
| **All livebirths** |  |  |  |
| Mean days of facility stay for the neonates who survived the neonatal period* | 1.37 days | 0.66 days | 3.69 days |
| Mean days of facility stay for the neonates who died during the neonatal period^†^ | 1.84 days | 0.84 days | 3.62 days |
| **0-2 days deaths** |  |  |  |
| Total neonates who died | 196 | 120 | 76 |
| Among the above, neonates who were discharged alive from facility after birth^‡^ | 112 (57.1%) | 75 (62.5%) | 37 (48.7%) |
| Mean days of facility stay for the above neonates who were discharged alive and died later^§^ | 1.21 days | 0.58 days | 2.46 days |
| **3-7 days deaths** |  |  |  |
| Total neonates who died | 76 | 54 | 22 |
| Among the above, neonates who were discharged alive from facility after birth^#^ | 65 (85.5%) | 51 (94.4%) | 14 (63.6%) |
| Mean days of facility stay for the above neonates who were discharged alive and died later** | 1.16 days | 0.61 days | 3.31 days |
| **8-27 days deaths** |  |  |  |
| Total neonates who died | 65 | 42 | 23 |
| Among the above, neonates who were discharged alive from facility after birth^††^ | 60 (92.3%) | 38 (90.5%) | 22 (95.7%) |
| Mean days of facility stay for the above neonates who were discharged alive and died later^‡‡^ | 2.51 days | 1.03 days | 5.00 days |

*t test value for public versus private facility mean days: 57.7, p<0.001

^†^t test value for public versus private facility mean days: -8.6, p<0.001

^‡^z test value for public versus private facility mean days: 1.9, p=0.057

^§^t test value for public versus private facility mean days: -4.0, p<0.001

^#^z test value for public versus private facility mean days: 3.5, p<0.001

**t test value for public versus private facility mean days: -5.7, p<0.001

^††^ z test value for public versus private facility mean days: -0.8, p=0.453

^‡‡^ t test value for public versus private facility mean days: -3.8, p<0.001
