# Supplementary material for: Distinct mortality patterns at 0–2 days versus the remaining neonatal period: results from population-based assessment in the Indian state of Bihar
Source: BMC Med. 2019 Jul 19;17:140. doi: 10.1186/s12916-019-1372-z (PMC6639919; doi:10.1186/s12916-019-1372-z)
Supplement: Supplementary file 3 — Figure S1. Distribution of illness symptoms (not mutually exclusive) and treatment sought for that symptom among newborns who survived 3 days or more in the Indian state of Bihar. (DOCX 23 kb) [file 12916_2019_1372_MOESM3_ESM.docx]

**Additional Figure 1**. Distribution of illness symptoms (not mutually exclusive) and treatment sought for that symptom among newborns who survived 3 days or more in the Indian state of Bihar.
