# Supplementary material for: Distinct mortality patterns at 0–2 days versus the remaining neonatal period: results from population-based assessment in the Indian state of Bihar
Source: BMC Med. 2019 Jul 19;17:140. doi: 10.1186/s12916-019-1372-z (PMC6639919; doi:10.1186/s12916-019-1372-z)

**Additional Figure 2**. Distribution of causes of death using verbal autopsy interviews by place of delivery for the three age sub-groups in the Indian state of Bihar.

| **0-2 days deaths** | | |
| --- | --- | --- |
|  |  |  |
| **3-7 days deaths** | | |
|  |  |  |
| **8-27 days deaths** | | |
|  |  |  |


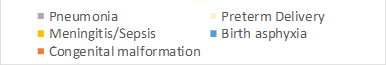

Supplement: Supplementary file 4 — Figure S2. Distribution of causes of death using verbal autopsy interviews by place of delivery for the three age sub-groups in the Indian state of Bihar. (DOCX 66 kb) [file 12916_2019_1372_MOESM4_ESM.docx]
